# Jun dimerization protein 2 controls hypoxia‐induced replicative senescence via both the p16^Ink4a^‐pRb and Arf‐p53 pathways

**DOI:** 10.1002/2211-5463.12325

**Published:** 2017-10-16

**Authors:** Koji Nakade, Chang‐Shen Lin, Xiao‐Yu Chen, Ming‐Ho Tsai, Kenly Wuputra, Zhi‐Wei Zhu, Jian‐Zhi Pan, Kazunari K. Yokoyama

**Affiliations:** ^1^ Gene Engineering Division RIKEN BioResource Center Tsukuba Japan; ^2^ Graduate Institute of Medicine Kaohsiung Medical University Taiwan; ^3^ Department of Biological Sciences National Sun Yat‐sen University Kaohsiung Taiwan; ^4^ Institute of Animal Husbandry and Veterinary ZheJiang Academy of Agricultural Sciences China; ^5^ Center for Stem Cell Research Kaohsiung Medical University Taiwan; ^6^ Center of Infectious Disease and Cancer Research Kaohsiung Medical University Taiwan; ^7^ Center for Environmental Medicine Kaohsiung Medical University Taiwan; ^8^ Faculty of Molecular Preventive Medicine Graduate School of Medicine The University of Tokyo Japan; ^9^ Faculty of Science and Engineering Tokushima Bunri University Sanuki Japan

**Keywords:** Arf, JDP2, p16^Ink4a^, p53, pRb, replicative senescence

## Abstract

The main regulators of replicative senescence in mice are p16^Ink4a^ and Arf, inhibitors of cell cycle progression. Jun dimerization protein 2 (JDP2)‐deficient mouse embryonic fibroblasts are resistant to replicative senescence through recruitment of the Polycomb repressive complexes 1 and 2 to the promoter of the gene that encodes p16^Ink4a^ and inhibits the methylation of lysine 27 of the histone H3 locus. However, whether or not JDP2 is able to regulate the chromatin signaling of either p16^Ink4a^‐pRb or Arf‐p53, or both, in response to oxidative stress remains elusive. Thus, this study sought to clarify this point. We demonstrated that the introduction of JDP2 leads to upregulation of p16^Ink4a^ and Arf and decreases cell proliferation in the presence of environmental (20% O_2_), but not in low (3% O_2_) oxygen. JDP2‐mediated growth suppression was inhibited by the downregulation of both p16^Ink4a^ and Arf. Conversely, the forced expression of p16^Ink4a^ or Arf inhibited cell growth even in the absence of JDP2. The downregulation of both the p53 and pRb pathways, but not each individually, was sufficient to block JDP2‐dependent growth inhibition. These data suggest that JDP2 induces p16^Ink4a^ and Arf by mediating signals from oxidative stress, resulting in cell cycle arrest via both the p16^Ink4a^‐pRb and Arf‐p53 pathways.

**Abbreviations**
EdU: 5‐ethynyl‐2′‐deoxyuridine
JDP2: Jun dimerization protein 2
MEFs: mouse embryonic fibroblasts
PRC1: Polycomb repressive complex 1
PRC2: Polycomb repressive complex 2
Wt: wild‐type

After several weeks in cell culture, primary cells stop proliferating and enter an irreversible growth arrest stage called replicative senescence. Replicative senescence involves processes that include the accumulation of oxidative stress, genotoxic stress, and telomere shortening. These stimuli finally induce the expression of p16^Ink4a^ and Arf. In the case of cultured mouse fibroblasts, cells undergo senescence even though they have long telomeres and high telomerase activity 1. The maintenance of telomere length remains important, as telomerase deficiency shortens the life span of mice and leads to premature aging 2, 3, 4. However, it seems likely that factors such as oxidative stress, rather than telomere shortening, contribute to the activation of the Ink4a/Arf locus in the case of mouse fibroblasts, based on the observation that mouse embryonic fibroblasts (MEFs) cultured in low (3%) oxygen can proliferate for longer periods without senescence 5. Transcription from the *Ink4a/Arf* locus is under complex control; p16^Ink4a^ and Arf respond independently to positive and negative signals, and the entire locus is epigenetically regulated. In young proliferating primary cells, the locus is transcriptionally silenced by the trimethylation of lysine 27 of histone H3 (H3K27). By contrast, the expression of p16^Ink4a^ and Arf increases in aged and senescent cells as a result of the loss of H3K27 trimethylation 6. The methylation of H3K27 and the silencing of the *p16* ^*Ink4a*^ */Arf* locus are mediated by the Polycomb repressive complexes 1 (PRC1) and 2 (PRC2). PRC1 and PRC2 form a complex at the *Ink4a/Arf* locus in young cells, and dissociate from this locus in aged cells. A proposed molecular mechanism of PRC‐mediated gene silencing is that Ezh2, a catalytic subunit of PRC2, trimethylates histone H3K27 7, which acts as a binding site for the CBX subunit of PRC1 8, 9. The Ring1B and Bmi1 subunits of PRC1 ubiquitylate histone H2A 10, 11, resulting in compaction of chromatin and inhibition of the elongation of RNA polymerase II 12. In aged and stressed cells, H3K27 trimethylation markers are lost because of the H3K27‐specific demethylase, Jumonji domain containing 3C 13, 14, and PRC1 dissociates from the p16^Ink4a^ locus, resulting in transcriptional activation by activators that include Ets1 and/or Ets2 15. p16^Ink4a^ binds cdk4/6 and changes its conformation, which prevents the phosphorylation of pRb by the cdk4/6–cyclin D complex. Thus, pRb‐bound E2F fails to activate genes that are essential for cell cycle progression, such as cyclin E1. Arf is an inhibitor of mouse double minute 2 homolog, which, in turn, mediates the degradation of p53. Therefore, the expression of Arf stabilizes p53 and activates its downstream cell cycle inhibitors, including p21^Cip/Waf1^. In brief, p16^Ink4a^ and Arf inhibit cell proliferation via pRb‐ and p53‐dependent pathways, respectively.

The Jun dimerization protein 2 (JDP2) is a chromatin‐remodeling factor 16, which has been implicated in various biological processes, including proliferation, differentiation, and apoptosis 17, 18, 19, 20, 21, 22, 23. We reported previously that MEFs from *Jdp2‐*knockout mice (*Jdp2* ^*−/−*^ MEF) proliferate for longer periods than wild‐type (Wt) MEFs. Subsequently, we found that the expression of p16^Ink4a^ and Arf was downregulated in *Jdp2* ^−/−^ MEFs 24.

In this study, we showed that overexpression of JDP2 led to the upregulation of p16^Ink4a^ and Arf. We downregulated p16^Ink4a^ and Arf using a lentiviral shRNA, to assess whether the inhibition of cell proliferation might be dependent on p16^Ink4a^ and Arf, and found that the overexpression of JDP2 failed to induce cell cycle arrest in the p16^Ink4a^/Arf‐deficient condition. Interestingly, JDP2 did not inhibit cell cycle progression in hypoxia (3% O_2_), but did so at environmental oxygen concentration (20% O_2_). In addition, we demonstrated that the downregulation of both p53 and pRb, but not of each of them individually, suppressed JDP2‐dependent growth inhibition. Taken together, these results suggest that JDP2 mediates signals that arise from environmental oxidative stress, induces the expression of p16^Ink4a^ and Arf, and inhibits cell cycle progression via both the p53 and pRb pathways.

## Materials and methods

### Lentiviral vectors

The plko‐based lentiviral shRNA vectors were purchased from the following manufacturers: shp16/Arf: TRCN0000077813 (Open Biosystems, Dharmacon, Marlborough, MA, USA); shp53: TRCN0000012359 (Open Biosystems); shpRb: TRCN0000042543 (Sigma‐Aldrich, Merck, Darmstadt, Germany); and shJDP2: TRCN0000081977 (Sigma‐Aldrich). shRNA was evaluated by infecting each lentivirus into MEF followed by the detection of the respective genes by real‐time PCR, as shown in Fig. S1. For the lentiviral expression vectors, cDNA for p16^Ink4a^ and Arf were generated from the total RNA of MEFs by RT‐PCR. The lentiviral expression vectors were constructed by inserting the open reading frame of JDP2 (for CSII‐JDP2), p16^Ink4a^ (for CSII‐p16^Ink4a^), or Arf (for CSII‐Arf) into CSII‐CMV‐MCS (RDB04377, RIKEN BioResource Center, Tsukuba, Japan). The lentiviruses were produced by cotransfecting 293T cells with the expression vector or shRNA vector together with packaging vectors [pCAG‐HIVgp (RDB04394, RIKEN BioResource Center) and pCMV‐VSV‐G‐RSV‐Rev (RDB04393, RIKEN BioResource Center)] followed by harvesting 3 days after transfection.

### Real‐time RT‐PCR

Total RNA was extracted using TRIzol reagent (15596‐026; Life Technologies, Carlsbad, CA, USA) according to the manufacturer's instructions. Reverse transcription was performed using Superscript III (18080‐051; Thermo Fisher Scientific Inc., Waltham, MA, USA) and the reverse primers specific for each gene, as described below. The real‐time PCR was performed using the Power SYBR Green Master Mix (4367659; Thermo Fisher Scientific Inc.) and the following primers: p16^Ink4a^, 5′‐gagtccgctgcagacagact‐3′ and 5′‐ccaggcatcgcgcacatcca‐3′; Arf, 5′‐agttcgtgcgatcccggaga‐3′ and 5′‐ccaggcatcgcgcacatcca‐3′; Jdp2, 5′‐cgctgacatccgcaacattg‐3′ and 5′‐catctggctgcagcgacttt‐3′; GAPDH, 5′‐ccacttgaagggtggagcca‐3′ and 5′‐tcatggatgaccttggccag‐3′; p53, 5′‐acagcacatgacggaggtcgtgaga‐3′ and 5′‐gcaggagctattacacatgtacttg‐3′; pRb, 5′‐acttggagtccgattgtattaccg‐3′ and 5′‐caacttcaagagcacaggccagta‐3′. PCR condition was as follows: initiation step for 10 min at 95 °C, followed by 40 thermal cycles of 95 °C for 10 s, 62 °C for 15 s, and 72 °C for 15 s using an ABI Prism 7700 Sequence Detection System (Thermo Fisher Scientific Inc.). Each value was normalized by dividing it by that of GAPDH, and results are shown as the ‘relative expression level’. The presented data are means of triplicate experiments.

### 5‐Ethynyl‐2′‐deoxyuridine (EdU) incorporation assay

MEFs cultured in a 3% O_2_/5% CO_2_ incubator were infected with the lentivirus for shRNA and/or that for gene expression at an multiplicity of infection (MOI) of 3. Two days later, the infected cells were selected in the presence of 10 μg·mL^−1^ of puromycin (for the shRNA expression vector) and/or 10 μg·mL^−1^ of blasticidin (for the protein expression vector) and incubated for at least 2 days in the presence of 3% O_2_ in order to eliminate uninfected cells. After selection, MEFs were transferred to a 20% O_2_/5% CO_2_ incubator and cultured for the indicated number of days. The cell proliferation assay was performed using a Click‐iT EdU Alexa Fluor Imaging Kit (C10337; Thermo Fisher Scientific Inc.) according to the manufacturer's instructions. Briefly, MEFs were replated into a 24‐well plate at various cell densities. On the next day, wells with 300–600 cells per 3.74 mm^2^ were used for the following EdU assay. A EdU solution was added and MEF cells were incubated for 8–14 h, to allow the incorporation of EdU into newly synthesized DNA. The incubation time for EdU incorporation was optimized by considering the doubling times of MEF, which were calculated as pilot experiments as described elsewhere 24, For example, in 5‐ to 8‐day cultured cells, the doubling time was 92 and 55 h for Wt and *Jdp2* ^*−/−*^ MEF, respectively; however, in 12‐ to 16‐day cultured cells, it was 109 and 61 h for Wt and *Jdp2* ^*−/−*^ MEF, respectively. In the case of the hypoxia‐cultured condition (3% O_2_), the doubling time was 57 and 49 h, respectively. After the direct fixation and permeabilization of the cells on the plate, proliferating cells were visualized by Alexa Fluor 488 or 594, which bound covalently to the incorporated EdU. After the EdU reaction, whole cells were stained with Hoechst 33342 dye. Proliferating (EdU‐positive) cells and whole cells were photographed under fluorescence microscopy (IX70 with UPIanFL4x/0.13; Olympus, Tokyo, Japan, fitted with a CCD Camera DFC300FX; Leica, Münich, Germany), and cells were counted using cell count version 1.1.7 software (vetSG, http://www.vector.co.jp/download/file/win95/art/fh454491.html). The average percentage of EdU‐positive cells was obtained from counting at least five individual images and more than three repeated experiments.

### ChIP assay

*Jdp2* ^*−/−*^ MEF cells cultured in a 3% O_2_/5% CO_2_ incubator were infected with the lentivirus for the expression of JDP2 (CSII‐JDP2) or the corresponding empty vector (CSII‐CMV‐MCS) at an MOI of 3. After 2 days’ infection, the cells were selected in the presence of 10 μg·mL^−1^ of blasticidin for 3 days and were further cultured for 1 week in a 20% O_2_/5% CO_2_ or 3% O_2_/5% CO_2_ incubator. The cells were used for ChIP assay using an anti‐histone H3 trimethyl‐H3K27 (6002; AbCam, Cambridge, MA, USA) and a Lowcell ChIP Kit (311‐80761; Diagenode, Denville, NJ, USA), according to the manufacturer's instructions. The precipitated DNA was analyzed by real‐time PCR using a Power SYBR Green Master Mix and a set of the following primers: for the p16^Ink4a^ locus: 5′‐gacccactggtcacacgact‐3′ and 5′‐tacccgactgcagatgggac‐3′; and for the Arf locus: 5′‐agttcgtgcgatcccggaga‐3′ and 5′‐ gcagcttcggagggcctttc‐3′. The PCR condition was as follows: initiation for 10 min at 95 °C, followed by five thermal cycles of 95 °C for 15 s, 62 °C for 4 min and 40 thermal cycles of 95 °C for 10 s, 62 °C for 30 s, 72 °C for 15 s using an ABI Prism 7700 Sequence Detection System. Each value was standardized by dividing by that of the input DNA and is shown as the ‘relative DNA amount’. The presented data are means of triplicate experiments.

### AlamarBlue assay

Cell proliferation was examined by the methods with AlamarBlue reagent assays according to the manufacturers’ instructions. The cells in the 96‐well plate were cultured for 1 or 4 days in the presence of 100 mL of Dulbecco's modified Eagle's medium supplemented with 10% FBS. Subsequently, 10 mL per well of AlamarBlue solution (DAL1100; Thermo Fisher Scientific Inc.) was added and further incubated for 2 h. The absorbance of each well was measured at 595 nm, and the cell numbers in the well were calculated. The standard deviation (SD) for the calculation of cell number was generated by pilot experiments using known samples. The division times (fold) from days 11 to 14 are shown in Fig. S2.

### Statistical analysis

The quantitative variables are presented as the mean ± SD. The significance of differences was determined using an unpaired two‐tailed Student's *t*‐test. Differences with a *P* value < 0.05 were considered significant.

## Results and Discussion

### Comparative cell proliferation and expression of p16^Ink4a^ and Arf in the normoxic and hypoxic conditions

**Figure 1:**
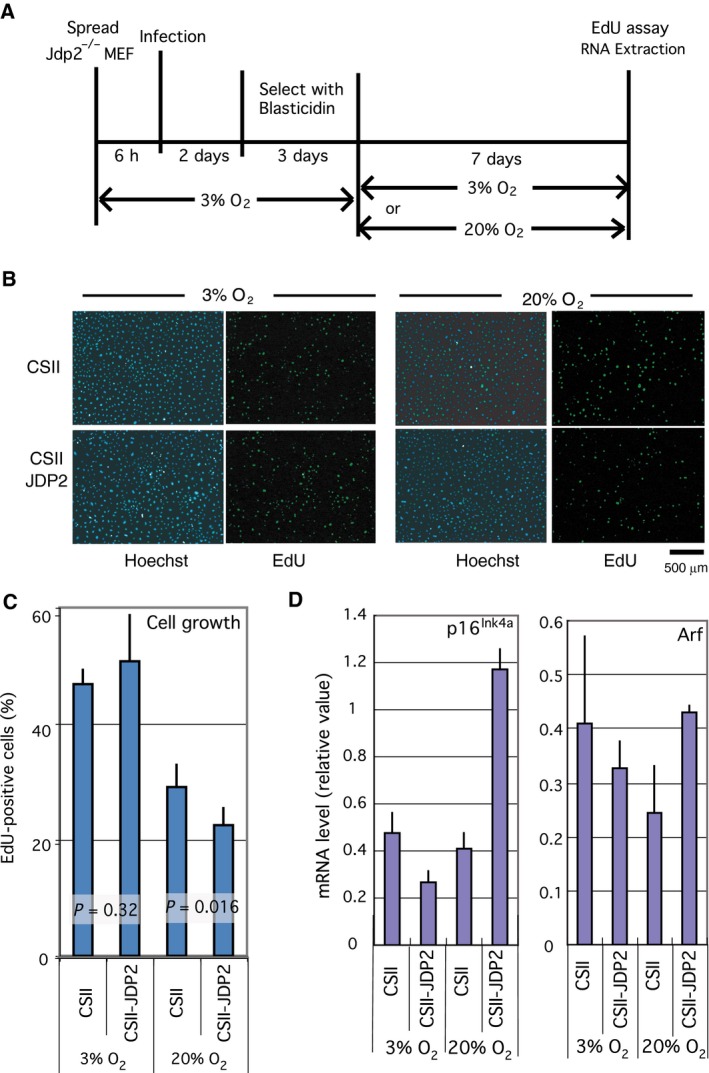
Inhibition of cell proliferation by JDP2 is oxidative stress dependent. (A) Schematic diagram showing the details of the assay conditions. *Jdp2* ^*−/−*^ MEFs cultured in the low oxygen concentration (3% O_2_) were infected with a lentivirus expressing JDP2 (CSII‐JDP2) or the empty vector (CSII). The infected cells were selected using blasticidin in low oxygen concentration, then cultured in the environmental (20% O_2_) or low oxygen concentrations for 7 days. The proliferation rate was analyzed using a EdU incorporation assay. (B) Cell proliferation was assayed via EdU incorporation. Cells were cultured in the presence of EdU for 14 h. Whole cells and proliferating cells were stained with Hoechst 33342 dye (right panel) and a Click‐iT EdU Alexa Fluor Imaging Kit (left panel), respectively. We performed nine different experiments. Scale bar = 500 μm. (C) Rate of growing cells in the EdU incorporation assay. The number of EdU‐positive and Hoechst‐stained cells was counted, and the percentages of growing (EdU‐positive) cells and their *P*‐values were calculated. The data stemmed from more than five different images. The numbers of counted cells are shown in Table S1. (D) Expression of the mRNA for p16^Ink4a^ (left) and Arf (right). Total RNA was extracted from cells cultured for 7 days in environmental (20% O_2_) or low oxygen (3% O_2_) concentrations. The mRNA levels were analyzed by real‐time RT‐PCR using specific primers. Error bars in each graph represent the SD of the data.

To evaluate the effect of JDP2 on cell proliferation at environmental oxygen concentration (20% O_2_), we generated JDP2 stable transformants by infecting *Jdp2* ^*−/−*^ MEFs with a lentiviral JDP2 expression vector. After selecting the transformants based on blasticidin resistance in the low oxygen condition (3% O_2_), the cells were further cultured in 20% or 3% O_2_ for 1 week (Fig. 1A). The percentage of growing cells was analyzed by the EdU incorporation assay. The growth of JDP2 transformants was inhibited compared with that of the control (empty vector) transformants after 1 week of cell culture in 20% O_2_ (*P* = 0.016; Fig. 1B,C). By contrast, no significant difference in proliferation between JDP2 and the empty vector transformants was observed when the cells were cultured in 3% O_2_ (*P* = 0.32; Fig. 1B,C). To confirm these results of EdU incorporation using an alternative approach, we also performed an AlamarBlue's assay. We compared the growth rates of the JDP2 and empty vector transformants from day 11 to day 14 after exposure to environmental O_2_ concentration. The result also showed that the cell growth in the presence of JDP2 was inhibited compared with that of the empty vector control under the environmental O_2_ concentration, but not under low oxygen concentration (Fig. S2). We analyzed the expression of the mRNA for p16^Ink4a^ and Arf by real‐time RT‐PCR. We found that the expression of p16^Ink4a^ and Arf was elevated in the JDP2 transformants compared with that of the empty vector transformants cultured in 20% O_2_ (Fig. 1D), but not in 3% O_2_ (Fig. 1D). The balancing of the effect of p16^Ink4a^ and Arf decided the rate of proliferation in this case. Taken together, these data indicate that both the overexpression of JDP2 and environmental oxidative stress are required for the induction of JDP2‐dependent growth inhibition.

### JDP2 plays a role in an oxidative stress‐dependent inhibition of cell growth

**Figure 2:**
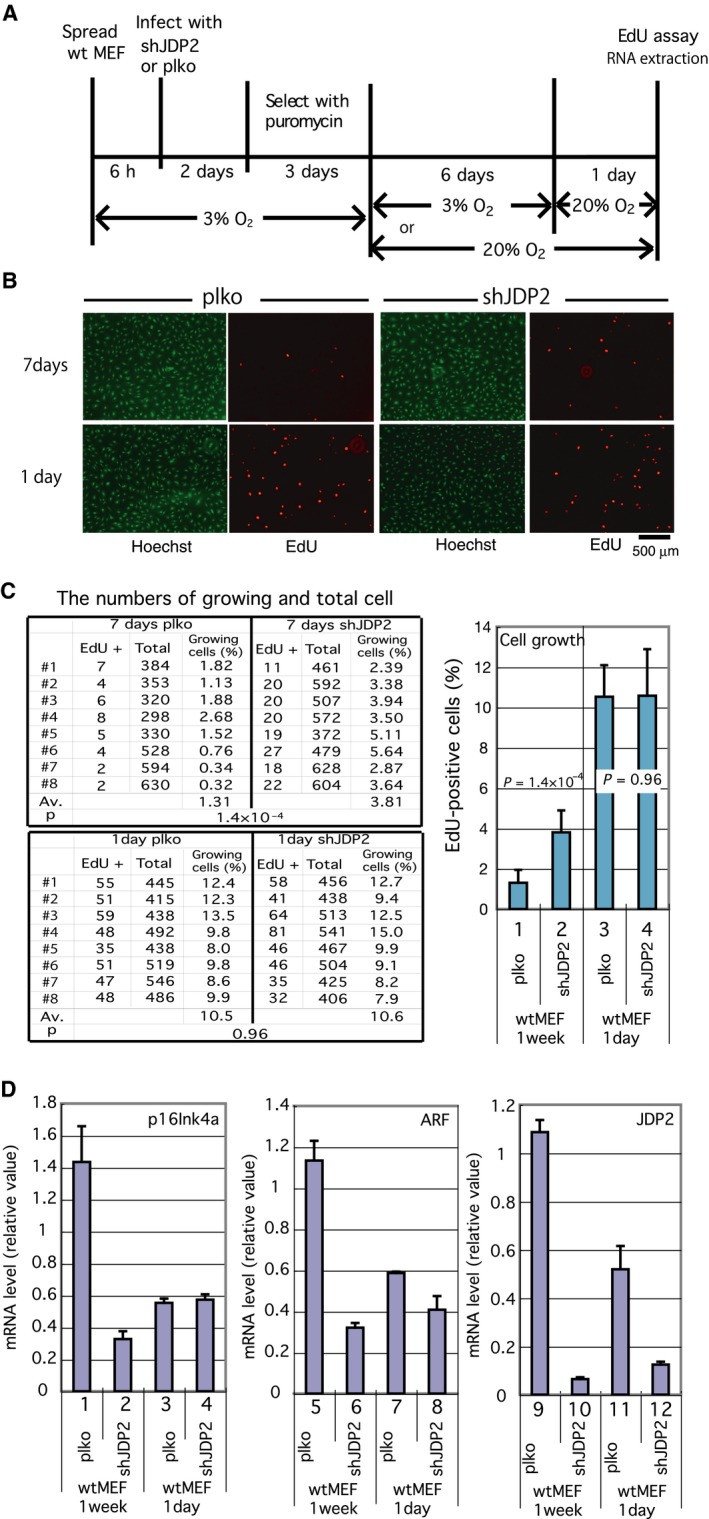
Downregulation of JDP2 suppressed the growth arrest induced by oxidative stress. (A) Schematic diagram showing details of the assay conditions. Wt MEFs cultured in low oxygen concentration (3% O_2_) were infected with a lentivirus expressing shRNA for JDP2 (shJDP2) or the empty vector (plko). The infected cells were selected by puromycin in low oxygen concentration, then cultured in the environmental (20% O_2_) or in the low oxygen concentration for 6 days. The cells were further cultured for 1 day in environmental oxygen concentration, and the proliferation rate was analyzed using an EdU incorporation assay. (B) Effect of shJDP2 on the proliferation activity of MEFs. The cells were cultured in the presence of EdU for 7 h. Whole cells and proliferating cells were visualized using Hoechst 33342 dye and a Click‐iT EdU Alexa Fluor Imaging Kit, respectively. We performed eight different experiments. Scale bar = 500 μm. (C) Effect of shJDP2 on cell growth of MEFs cultured for 1 and 7 days. The percentages of growing (EdU‐positive) cells against whole (Hoechst 33342‐stained) cells and the corresponding *P*‐values were calculated. We performed eight different experiments. The numbers of counted cells are shown in Table S1. (D) Expression of the mRNA for p16^Ink4a^ (left), Arf (middle), and JDP2 (right). Total RNA was extracted from the cells at the same time for the cell proliferation assay. The mRNA levels were analyzed by real‐time RT‐PCR using specific primers. Error bars in each graph represent the SD of the data.

Next, we examined whether endogenous JDP2 functions as an oxidative stress‐dependent inhibitor of cell growth because our previous study demonstrated that endogenous JDP2 was elevated in 20% O_2_ in comparison with that in 3% O_2_ 24. We generated transformants in which JDP2 was downregulated by infecting Wt MEFs with a lentiviral shRNA vector for JDP2 (shJDP2; Fig. 2A). After 1 week of cell culture in 20% O_2_, the shJDP2 transformants exhibited a higher rate of proliferation (*P* = 5.4 × 10^−4^; Fig. 2B,C). Conversely, after 1 day of culture in 20% O_2_ with a prior 6‐day treatment of cells under 3% O_2_, no significant differences in cell proliferation were observed (*P* = 0.82). Similar results were obtained from the EdU assay using different shRNA against JDP2 (TRCN0000081973; Fig. S1). The result of the experiment using this alternative shJDP2 demonstrated that, after 27 days of cell culture in 20% O_2_, the higher rate of cell growth of shJDP2 transformants was more significant (*P* = 1 × 10^−4^) than that of 7 days (*P* = 2.8 × 10^−3^) and no significant change was observed after 1 day (*P* = 0.71; Fig. S3). These data suggest that the effect of shJDP2 was specific. The EdU‐positive cells were increased by this treatment as compared with that of 1‐week culture of cells in 20% O_2_, and the endogenous expression of JDP did not reach the control level of plko transformants after 1 week's culture of cells in 20% O_2_ (Fig. 2C,D). These data indicate that a high level of endogenous JDP2 induces the inhibition of cell proliferation in response to the oxidative stress that accumulated for 1‐week culture.

We analyzed the expression of the mRNA for p16^Ink4a^ and Arf and observed a lower expression of both mRNA in the shJDP2 transformants after 1 week of cell culture in the environmental oxygen concentration (Fig. 2D). By contrast, no significant differences in the expression of p16^Ink4a^ were observed between the shJDP2 and empty vector transformants after 1 day of cell culture. However, Arf level in shJDP2 transformants was slightly lower than that in the control vector transformants. Both the p16^Ink4a^ and Arf mRNA levels were lower in 1‐day normoxia condition than that in 7 days (Fig. 2D), which reflected the difference in EdU‐positive cells between the two conditions (Fig. 2C).

### Effects of knockdown of p16^Ink4a^ and Arf on cell growth

**Figure 3:**
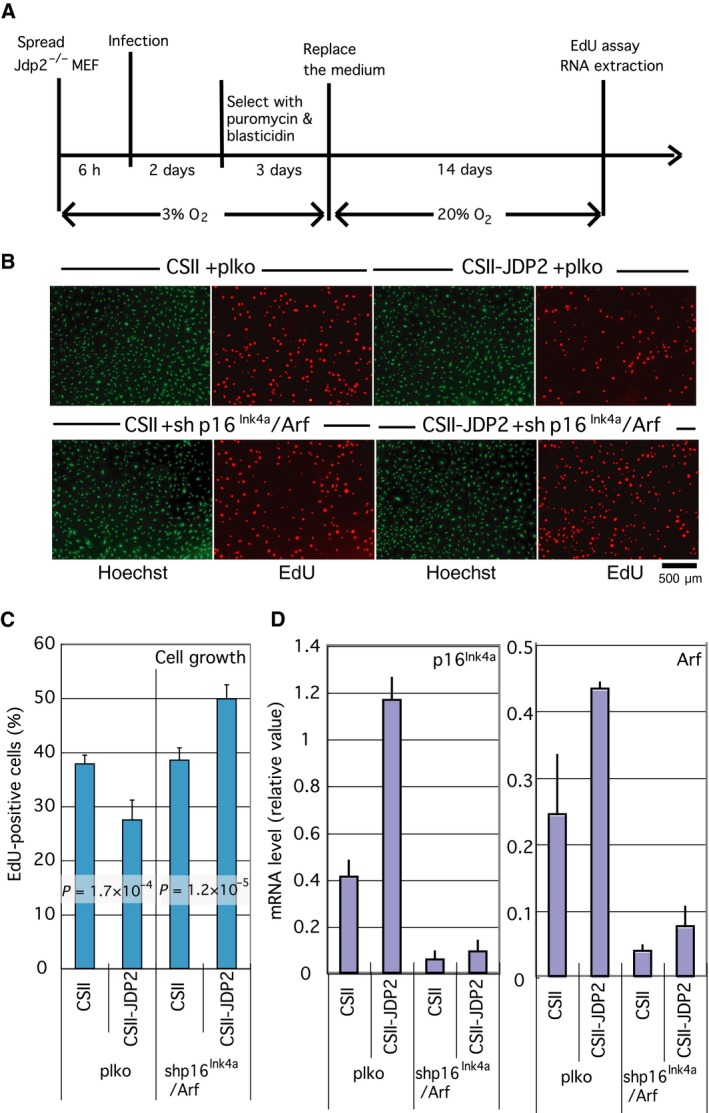
Inhibition of cell proliferation by JDP2 is p16^Ink4a^/Arf dependent. (A) Schematic diagram showing details of the assay conditions. *Jdp2* ^*−/−*^ MEF cultured in low oxygen (3% O_2_) concentration were coinfected with CSII (empty vector) or CSII‐JDP2 and plko (empty vector) or shp16^Ink4a^/Arf (which expresses shRNA for both p16^Ink4a^ and Arf). The infected cells were selected using blasticidin and puromycin in low oxygen for 3 days, then cultured in environmental oxygen concentration (20% O2) for 14 days. The rate of cell proliferation was analyzed via a EdU incorporation assay. (B) Effect of JDP2 in p16^Ink4a^/Arf downregulated MEF cells on cell proliferation. Cells cultured in the indicated condition were further labeled in the presence of EdU, for 16 h. Whole cells and proliferating cells were visualized using Hoechst 33342 dye and a Click‐iT EdU Alexa Fluor Imaging Kit, respectively. Scale bar = 500 μm. (C) Effect of expression of JDP2 on the cell proliferation in MEFs. The number of whole (Hoechst 333242‐stained) cells and growing (EdU‐positive) cells was counted. The percentages of growing cells against whole cells and their *P*‐values were calculated. We performed at least six different experiments. The numbers of counted cells are shown in Table S1. (D) Expression of the mRNA for p16^Ink4a^ (left) and Arf (right). At the same time, total RNA was extracted from the cells for the cell proliferation assay. The mRNA levels were analyzed by real‐time RT‐PCR using specific primers. Error bars in each graph represent the SD of the data.

Based on these results, we hypothesized that JDP2 mediates the signal from oxidative stress and increases the expression of cell cycle inhibitors, including p16^Ink4a^ and Arf. To substantiate this hypothesis, we generated transformants in which p16^Ink4a^ and Arf were downregulated by shRNA and analyzed the effect of JDP2 on cell proliferation after 2 weeks of cell culture in 20% O_2_ (Fig. 3A). In the presence of JDP2, the expression of the p16^Ink4a^ and Arf mRNA was increased (Fig. 3D), and cell proliferation was inhibited (*P* = 1.7 × 10^−4^; Fig. 3B,C). In contrast, cell growth was promoted rather than inhibited (*P* = 1.2 × 10^−5^) in the shp16^Ink4a/^Arf transformants, in which the mRNA for p16^Ink4a^ and Arf were downregulated.

These results suggest that JDP2 is an upstream positive regulator of p16^Ink4a^ and Arf and that the presence of one or both proteins is essential for effective JDP2‐mediated growth inhibition. We also confirmed that the forced expression of p16^Ink4a^ and Arf inhibited cell proliferation in both *Jdp2* ^*−/−*^ and Wt MEFs (Fig. S4A). Forced expression of p16^Ink4a^ did not increase the expression of JDP2 in Wt MEFs (Fig. S4B). These data suggest that JDP2 is neither a downstream factor in the signal transduction pathway nor a transcriptional target of p16^Ink4a^. Interestingly, the forced expression of Arf decreased the expression of JDP2; thus, a negative feedback loop depending on the Arf‐p53 pathway might exist.

### Both p53 and pRb pathways are required for JDP2‐dependent growth suppression

**Figure 4:**
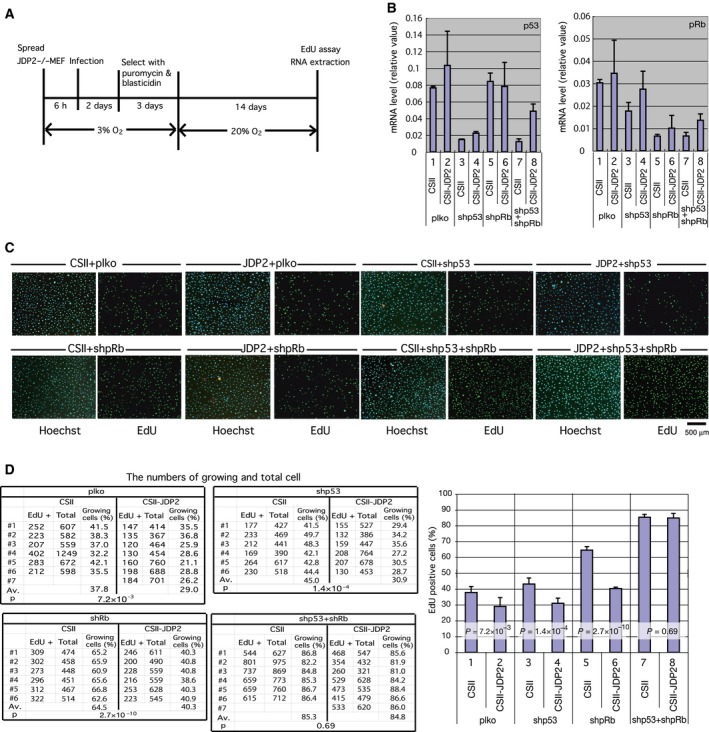
Inhibition of cell proliferation by JDP2 is dependent on both the p16^Ink4a^‐pRb and Arf‐p53 pathways. (A) Schematic diagram showing the details of assay conditions. *Jdp2* ^*−/−*^ MEFs cultured in the low oxygen condition were coinfected with CSII (empty vector) or CSII‐JDP2 and plko (empty vector) or shpRb and/or shp53 (which express shRNA for pRb and p53, respectively). The infected cells were selected by blasticidin and puromycin in low oxygen for 3 days and were then cultured in the environmental oxygen condition (20% O_2_) for 14 days. The rate of cell proliferation was analyzed using EdU incorporation assay. (B) Expression of mRNA for p53 (left) and pRb (right). Total RNA was extracted from the cells at the same time for the cell proliferation assay. The mRNA levels were analyzed by real‐time RT‐PCR using specific primers. (C) Effect of JDP2 on cell proliferation in pRb and/or p53 downregulated MEFs. The cells were cultured in the presence of EdU for 14 h. Whole cells and proliferating cells were visualized using Hoechst 33342 and a Click‐iT EdU Alexa Fluor Imaging Kit, respectively. We performed at least seven experiments. Scale bar = 500 μm. (D) The number of whole (Hoechst 33342‐stained) cells and growing (EdU‐positive) cells were counted. The percentage of growing cells against whole cells and their *P*‐values were calculated. The data were derived from at least six different images. The numbers of counted cells are shown in Table S1. Error bars in each graph represent the SD of the data.

We examined which pathway is essential for JDP2‐dependent cell cycle inhibition. As it is technically difficult to downregulate p16^Ink4a^ and Arf independently via shRNA, we downregulated their downstream targets, pRb and p53, respectively, via lentiviral shRNA and analyzed the effect of JDP2 on cell growth by cointroducing a JDP2 expression vector. The EdU incorporation assay was performed after 2 weeks of cell culture in 20% O_2_ (Fig. 4A). The downregulation of pRb and p53 was confirmed by real‐time RT‐PCR (Fig. 4B). The results of the cell proliferation assay demonstrated that the downregulation of either p53 or pRb was not sufficient for the blockage of the JDP2‐dependent growth inhibition (Fig. 4C,D). However, a deficiency in both p53 and pRb significantly inhibited the growth arrest observed in the presence of JDP2. These data demonstrate that both the Arf‐p53 and p16^Ink4a^‐pRb pathways are required for JDP2‐dependent inhibition of cell proliferation.

### JDP2 regulates the trimethylation of H3K27 at p16^Ink4a^ and Arf locus

**Figure 5:**
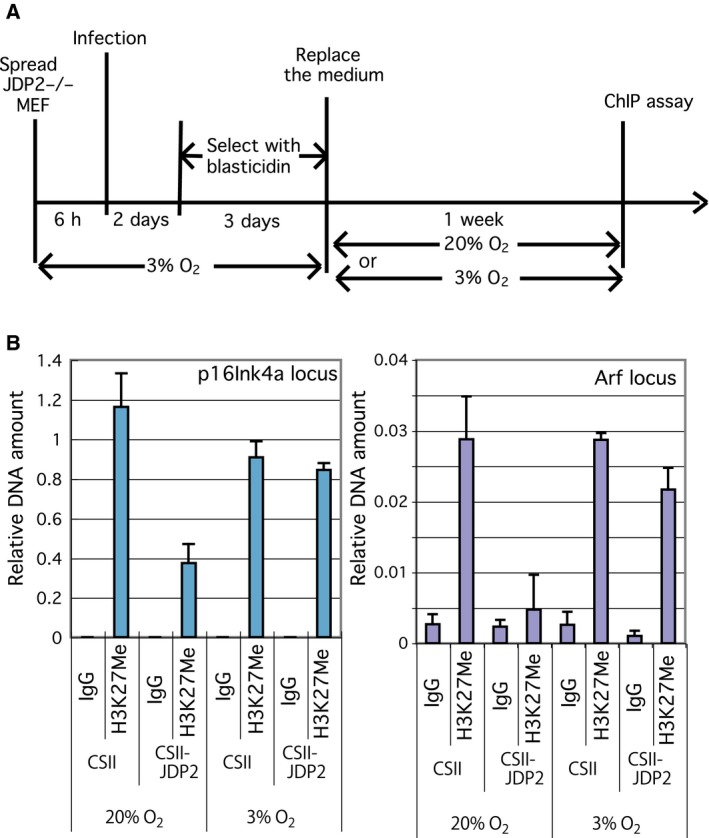
Effect of JDP2 on the trimethylation of histone H3K27 at the *p16* ^*Ink4a*^/*Arf* locus. (A) Schematic diagram showing the details of the assay conditions. *Jdp2* ^*−/−*^ MEF cultured in the low oxygen concentration were infected with CSII or CSII‐JDP2 and the transformants were selected using blasticidin for 3 days. The cells were cultured for 1 additional week in the environmental or low oxygen concentrations and their lysates were used in the ChIP assay. (B) Trimethylation of histone H3K27 at the p16^Ink4a^/Arf locus. The ChIP assay was performed using the cell lysates, which were treated as indicated in (A). The DNA fragments bound to the trimethyl‐H3K27 were immunoprecipitated using an anti‐trimethyl‐H3K27 antibody. The amount of DNA corresponding to the *p16* ^*Ink4a*^ */Arf* locus was assessed by real‐time RT‐PCR using specific primers. Each value was normalized to the amount of input DNA. Error bars in each graph represent the SD of the data.

As the expression of p16^Ink4a^ and Arf is epigenetically regulated by histone methylation, we analyzed the effect of JDP2 on the methylation of H3K27 in the *p16* ^*Ink4a*^ */Arf* locus. We performed a ChIP assay using an anti‐histone H3 trimethyl lysine 27 antibody and a cell extract of *Jdp2* ^*−/−*^ MEFs infected with the JDP2 expression vector (CSII‐JDP2) or the empty vector (CSII) and cultured cells for 1 week at the environmental or low oxygen concentration. The results of this experiment demonstrated that the extent of the trimethylation of lysine 27 in histone H3 at both the p16^Ink4a^ and Arf transcription regulatory sites was decreased in the presence of JDP2 under the environmental oxygen concentration (Fig. 5A,B). Under the low oxygen concentration, the trimethylation of lysine 27 remained at a higher level even in the presence of JDP2, suggesting that JDP2 affects the methylation of H3K27 only under the higher environmental oxygen conditions.

## Conclusion

**Figure 6:**
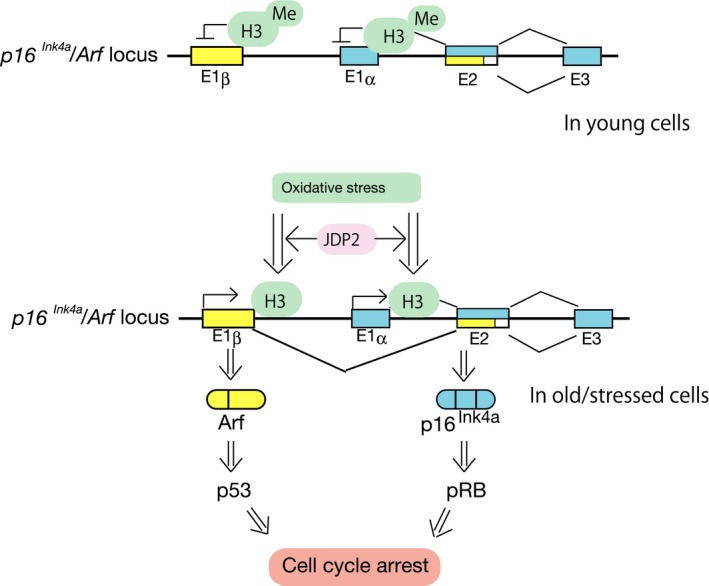
Schematic representation of the control mechanism of *p16* ^*Ink4a*^ */Arf* locus in young cells and in old/stressed cells. H3, histone H3; Me, methylated residues; E, exon.

Based on our findings, we propose the following model (Fig. 6). In young undamaged cells, the expression of p16^Ink4a^ and Arf is silenced because of the trimethylation of histone H3K27. The expression of JDP2 does not play a role in the absence of a stress signal. In old cells, the oxidative stress signal mediated by JDP2 induces the demethylation of histone H3 on the transcription regulation sites of both p16^Ink4a^ and Arf, resulting in the activation of the pRb and p53 pathways and the cell cycle arrest. Our findings demonstrate that JDP2 is essential for replicative senescence and is involved in both the p16^Ink4a^‐pRb and Arf‐p53 pathways for the inhibition of cell growth by mediating the signal from oxidative stress.

## Author contributions

KN, JZP, CSL, and KKY conceived and supervised the study; KN, JZP, and KKY designed experiments; KN, KW, MHT, and XYC performed experiments; JZP and KKY provided new tools and reagents; CSL, MHT, ZWZ, JZP, and KKY analyzed data; KN, CSL, JZP, and KKY drafted the manuscript.

## Supporting information


**Fig. S1.** Evaluation of the inhibitory activity of the lentiviral shRNA expression vectors.

**Fig. S2.** Effect of the forced expression of JDP2 on cell growth in *Jdp2* ^*−/−*^ MEFs using an AlamarBlue assay.

**Fig. S3.** Downregulation of JDP2 by shRNA targeting different site (TRCN0000081973) inhibited the growth arrest induced by oxidative stress.

**Fig. S4.** Forced expression of p16^Ink4a^ or Arf inhibits cell proliferation even in the absence of JDP2.

**Table S1.** Actual values of cell growth for Figs 1–4 are listed.
