# Supplementary figures and images for: Jun dimerization protein 2 controls hypoxia‐induced replicative senescence via both the p16Ink4a‐pRb and Arf‐p53 pathways
Source: FEBS Open Bio. 2017 Oct 16;7(11):1793–804. doi: 10.1002/2211-5463.12325 (PMC5666393; doi:10.1002/2211-5463.12325)

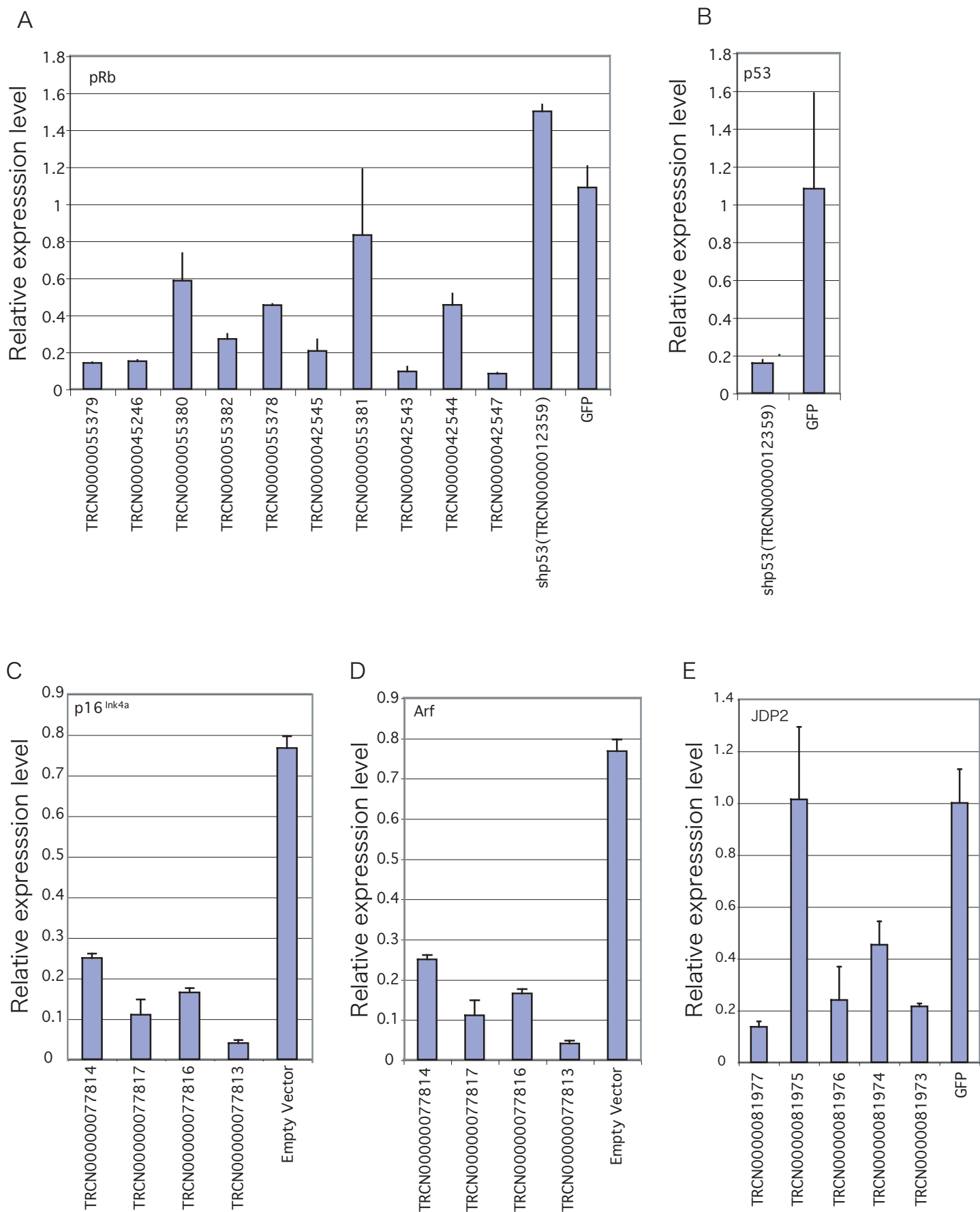

Supplement: Supplementary file 1 — Fig. S1. Evaluation of the inhibitory activity of the lentiviral shRNA expression vectors. [file FEB4-7-1793-s001.pdf]
