# Supplementary material for: Jun dimerization protein 2 controls hypoxia‐induced replicative senescence via both the p16Ink4a‐pRb and Arf‐p53 pathways
Source: FEBS Open Bio. 2017 Oct 16;7(11):1793–804. doi: 10.1002/2211-5463.12325 (PMC5666393; doi:10.1002/2211-5463.12325)
Supplement: Supplementary file 2 — Fig. S2. Effect of the forced expression of JDP2 on cell growth in Jdp2 −/− MEFs using an AlamarBlue assay. [file FEB4-7-1793-s002.pdf]

A

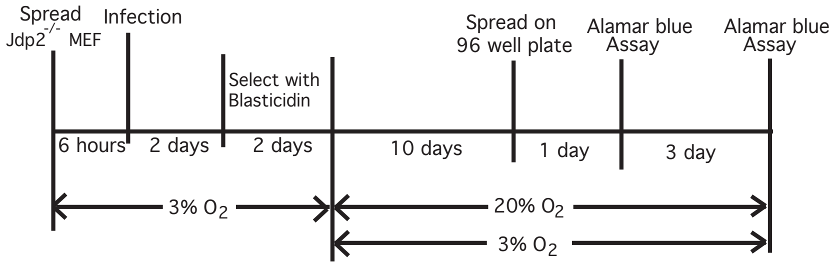

B

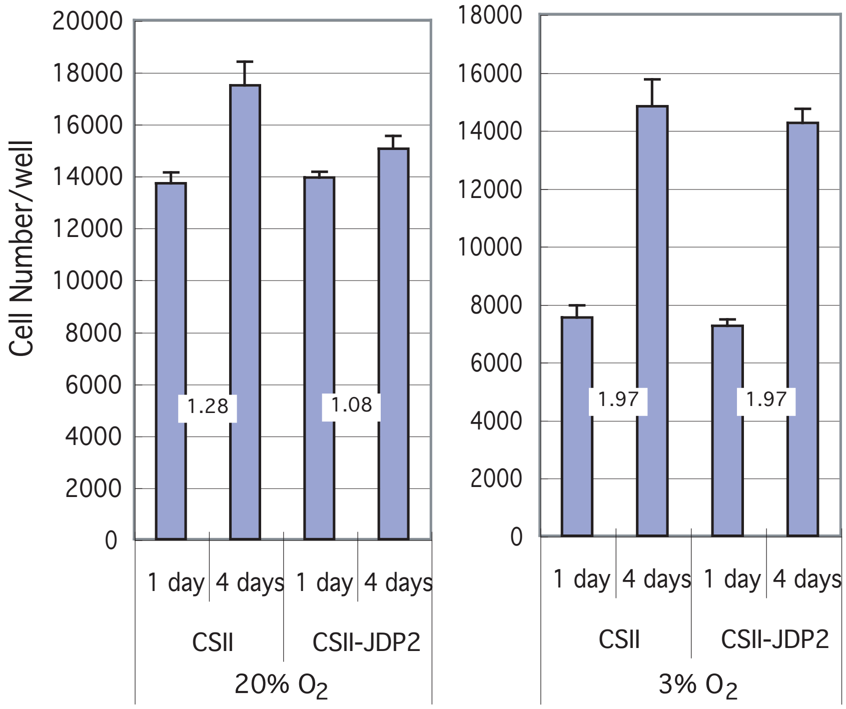

20% O_2		Average	SD
CSII	1 day	13700	420
	4 days	17500	910
CSII-JDP2	1 day	14000	220
	4 days	15100	480

3% O_2		Average	SD
CSII	1 day	7540	280
	4 days	14800	670
CSII-JDP2	1 day	7250	150
	4 days	14300	530

Nakade et al. Supplemental Fig. 2
