# Supplementary material for: Jun dimerization protein 2 controls hypoxia‐induced replicative senescence via both the p16Ink4a‐pRb and Arf‐p53 pathways
Source: FEBS Open Bio. 2017 Oct 16;7(11):1793–804. doi: 10.1002/2211-5463.12325 (PMC5666393; doi:10.1002/2211-5463.12325)
Supplement: Supplementary file 3 — Fig. S3. Downregulation of JDP2 by shRNA targeting different site (TRCN0000081973) inhibited the growth arrest induced by oxidative stress. [file FEB4-7-1793-s003.pdf]

A

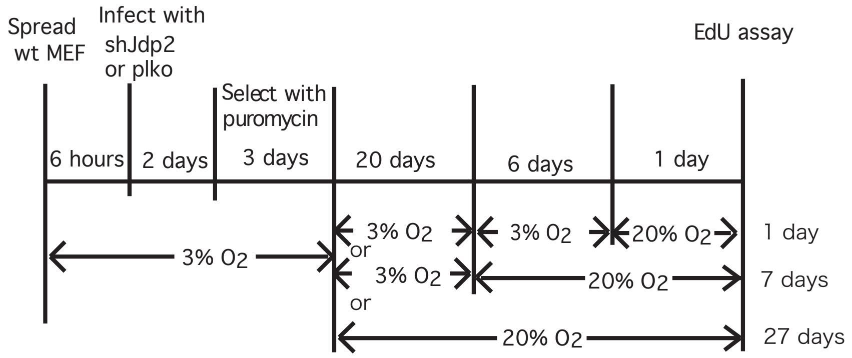


B

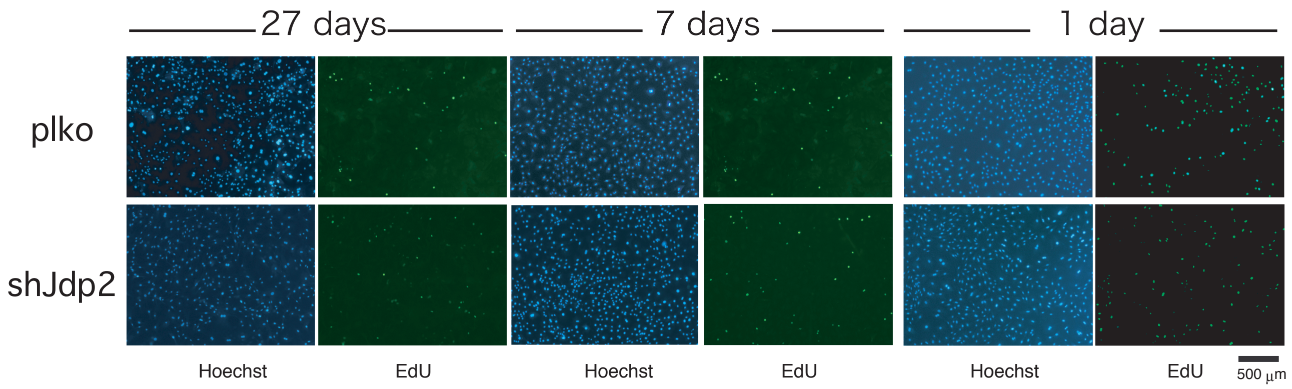


C

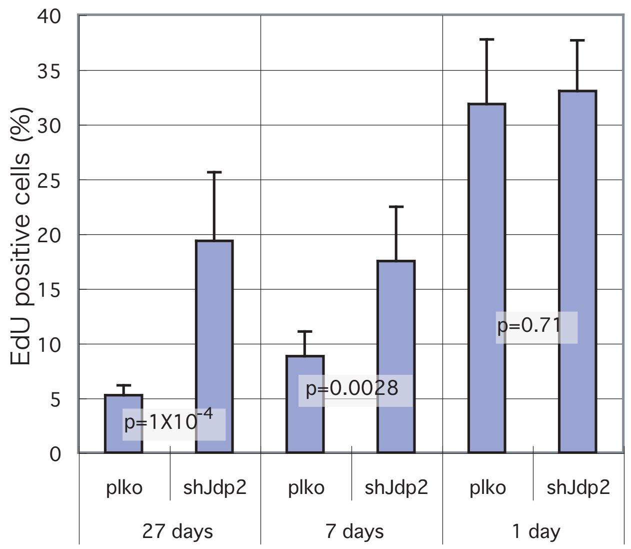


| | 27 day 20%O2+plko EdU + | Total | Growing cells(%) | 27 day 20%O2+shJdp2 EdU + | Total | Growing cells(%) |
|---|---|---|---|---|---|---|
| #1 | 38 | 657 | 5.8 | 109 | 428 | 25.5 |
| #2 | 52 | 780 | 6.7 | 87 | 744 | 11.7 |
| #3 | 34 | 636 | 5.3 | 111 | 429 | 25.9 |
| #4 | 35 | 707 | 5.0 | 68 | 548 | 12.4 |
| #5 | 17 | 405 | 4.2 | 90 | 488 | 18.4 |
| #6 | 34 | 766 | 4.4 | 97 | 436 | 22.2 |
| Av. | | | 5.2 | | | 19.4 |
| SD | | | 0.9 | | | 6.3 |
| p | | | $1X10^{-4}$ | | | |

| | 7 day 20%O2+plko EdU + | Total | Growing cells(%) | 7 day 20%O2+shJdp2 EdU + | Total | Growing cells(%) |
|---|---|---|---|---|---|---|
| #1 | 51 | 562 | 9.0 | 108 | 670 | 16.1 |
| #2 | 49 | 799 | 6.1 | 117 | 831 | 14.1 |
| #3 | 40 | 512 | 7.8 | 113 | 465 | 24.3 |
| #4 | 78 | 673 | 11.6 | 115 | 504 | 22.8 |
| #5 | 77 | 685 | 11.2 | 114 | 724 | 15.7 |
| #6 | 45 | 634 | 7.1 | 119 | 991 | 12.0 |
| Av. | | | 8.8 | | | 17.5 |
| SD | | | 2.2 | | | 4.9 |
| p | | | 0.0028 | | | |

| | 1 day 20%O2+plko EdU + | Total | Growing cells(%) | 1 day 20%O2+shJdp2 EdU + | Total | Growing cells(%) |
|---|---|---|---|---|---|---|
| #1 | 163 | 515 | 31.7 | 154 | 498 | 30.9 |
| #2 | 185 | 619 | 29.9 | 157 | 408 | 38.5 |
| #3 | 140 | 343 | 40.8 | 203 | 535 | 37.9 |
| #4 | 163 | 451 | 36.1 | 102 | 376 | 27.1 |
| #5 | 151 | 529 | 28.5 | 154 | 523 | 29.4 |
| #6 | 125 | 520 | 24.0 | 169 | 494 | 34.2 |
| Av. | | | 31.8 | | | 33.0 |
| SD | | | 5.9 | | | 4.6 |
| p | | | 0.71 | | | |

Nakade et al. Supplemental Fig. 3
