# Supplementary material for: Jun dimerization protein 2 controls hypoxia‐induced replicative senescence via both the p16Ink4a‐pRb and Arf‐p53 pathways
Source: FEBS Open Bio. 2017 Oct 16;7(11):1793–804. doi: 10.1002/2211-5463.12325 (PMC5666393; doi:10.1002/2211-5463.12325)
Supplement: Supplementary file 4 — Fig. S4. Forced expression of p16Ink4a or Arf inhibits cell proliferation even in the absence of JDP2. [file FEB4-7-1793-s004.pdf]

A

The numbers of growing and total cell

| | wt MEF | | | | | | | | |
|---|---|---|---|---|---|---|---|---|---|
| | CSII | | | CSII-p16Ink4a | | | CSII-Arf | | |
| | EdU + | Total | Growing cells(%) | EdU + | Total | Growing cells(%) | EdU + | Total | Growing cells(%) |
| #1 | 49 | 381 | 12.9 | 28 | 582 | 4.8 | 20 | 383 | 5.2 |
| #2 | 50 | 241 | 20.7 | 36 | 530 | 6.8 | 22 | 410 | 5.4 |
| #3 | 27 | 303 | 8.9 | 33 | 555 | 5.9 | 16 | 426 | 3.8 |
| #4 | 53 | 317 | 16.7 | 29 | 536 | 5.4 | 19 | 495 | 3.8 |
| #5 | 52 | 380 | 13.7 | 29 | 638 | 4.5 | 21 | 520 | 4.0 |
| #6 | 69 | 413 | 16.7 | 28 | 538 | 5.2 | 32 | 501 | 6.4 |
| Av. | | | 14.9 | | | 5.5 | | | 4.8 |
| SD | | | 4.1 | | | 0.8 | | | 1.1 |

| | Jdp2 -/- MEF | | | | | | | | |
|---|---|---|---|---|---|---|---|---|---|
| | CSII | | | CSII-p16Ink4a | | | CSII-Arf | | |
| | EdU + | Total | Growing cells(%) | EdU + | Total | Growing cells(%) | EdU + | Total | Growing cells(%) |
| #1 | 129 | 351 | 36.8 | 86 | 443 | 19.4 | 67 | 473 | 14.2 |
| #2 | 146 | 370 | 39.5 | 77 | 388 | 19.8 | 61 | 413 | 14.8 |
| #3 | 129 | 373 | 34.6 | 96 | 424 | 22.6 | 33 | 388 | 8.5 |
| #4 | 169 | 424 | 39.9 | 141 | 545 | 25.9 | 35 | 379 | 9.2 |
| #5 | 184 | 491 | 37.5 | 123 | 564 | 21.8 | 36 | 449 | 8.0 |
| #6 | 156 | 476 | 32.8 | 65 | 359 | 18.1 | 30 | 498 | 6.0 |
| Av. | | | 36.8 | | | 21.3 | | | 10.1 |
| SD | | | 2.8 | | | 2.8 | | | 3.5 |

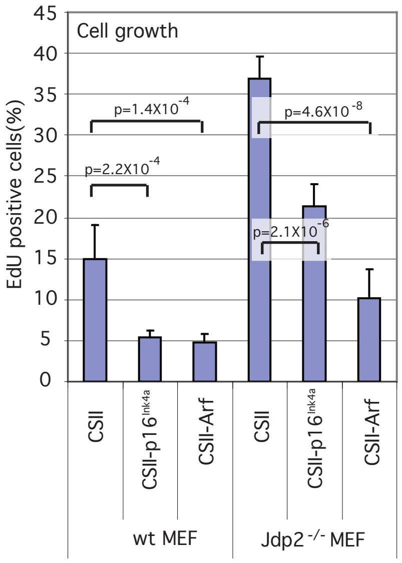


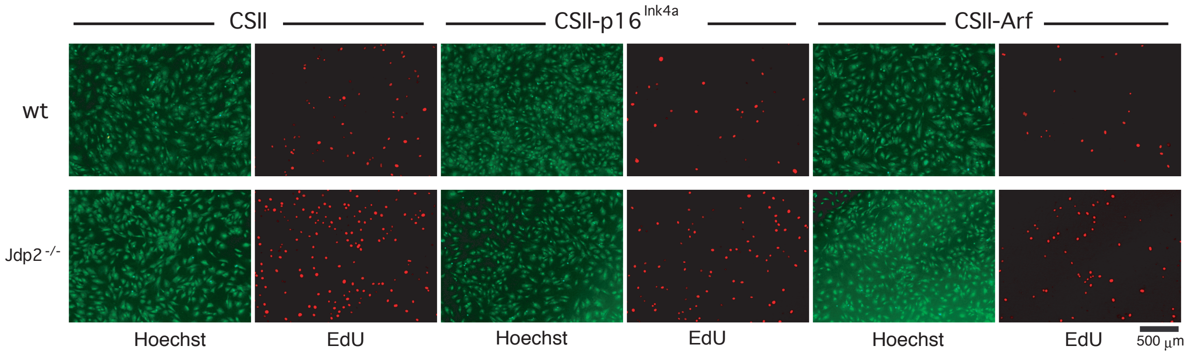


B

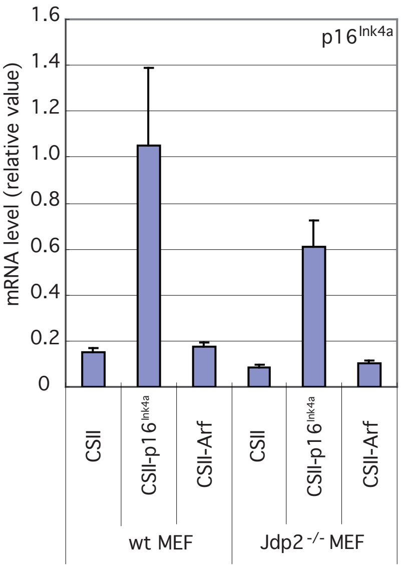


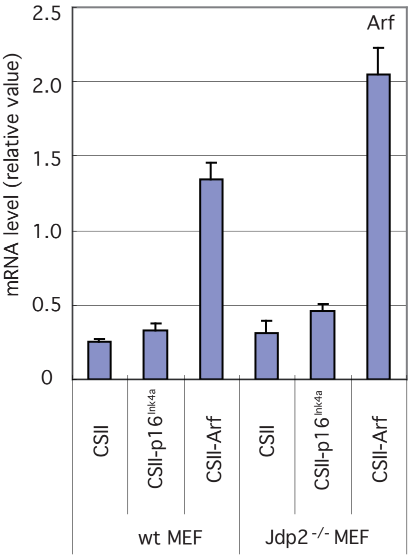


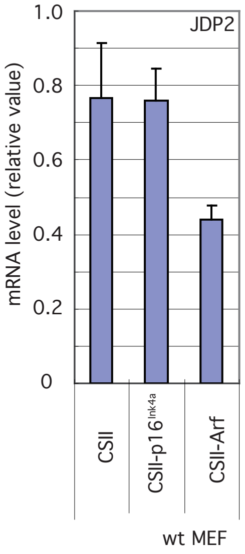


Nakade et al. Supplemental Fig. 4
