# Supplementary material for: Jun dimerization protein 2 controls hypoxia‐induced replicative senescence via both the p16Ink4a‐pRb and Arf‐p53 pathways
Source: FEBS Open Bio. 2017 Oct 16;7(11):1793–804. doi: 10.1002/2211-5463.12325 (PMC5666393; doi:10.1002/2211-5463.12325)
Supplement: Supplementary file 5 — Table S1. Actual values of cell growth for Figs 1–4 are listed. [file FEB4-7-1793-s005.pdf]

The numbers of growing and total cells for Figure 1

	3% O2 CSII			3% O2 CSII-JDP2		
	EdU +	Total	Growing cells(%)	EdU +	Total	Growing cells(%)
#1	238	502	47.4	313	552	56.7
#2	258	553	46.7	258	543	47.5
#3	293	677	43.3	347	598	58.0
#4	291	581	50.1	229	595	38.5
#5	285	612	46.6	283	535	52.9
Av.			46.8			50.7
SD			2.4			8.0
p	0.32					

	20% O2 CSII			20% O2 CSII-JDP2		
	EdU +	Total	Growing cells(%)	EdU +	Total	Growing cells(%)
#1	197	625	31.5	140	667	21.0
#2	182	596	30.5	152	635	23.9
#3	175	689	25.4	180	716	25.1
#4	301	907	33.2	157	643	24.4
#5	148	602	24.6	133	733	18.1
Av.			29.0			22.5
SD			3.8			2.9
p	0.016					

The numbers of growing and total cells for Figure 2

	7 days plko			7 days shJDP2		
	EdU +	Total	Growing cells(%)	EdU +	Total	Growing cells(%)
#1	7	384	1.82	11	461	2.39
#2	4	353	1.13	20	592	3.38
#3	6	320	1.88	20	507	3.94
#4	5	330	1.52	20	572	3.50
#5	4	528	0.76	27	479	5.64
#6	2	594	0.34	22	604	3.64
Av.			1.24			3.75
SD			0.61			1.07
p	$5.4X10^{-4}$					

	1day plko			1day shJDP2		
	EdU +	Total	Growing cells(%)	EdU +	Total	Growing cells(%)
#1	55	445	12.4	58	456	12.7
#2	59	438	13.5	41	438	9.4
#3	48	492	9.8	64	513	12.5
#4	35	438	8.0	46	467	9.9
#5	51	519	9.8	46	504	9.1
#6	48	486	9.9	35	425	8.2
Av.			10.5			10.3
SD			2.0			1.9
p	0.82					

The numbers of growing and total cells for Figure 3

plko						
	CSII			CSII-JDP2		
	EdU +	Total	Growing cells(%)	EdU +	Total	Growing cells(%)
#1	223	577	38.6	143	469	30.5
#2	243	623	39.0	112	470	23.8
#3	226	572	39.5	115	414	27.8
#4	197	557	35.4	127	439	28.9
#5	198	521	38.0	135	411	32.8
#6	230	632	36.4	87	387	22.5
Av.			37.8			27.7
SD			1.6			3.9
p	$1.7X10^{-4}$					

$shp16^{Ink4a}$ /Arf						
	CSII			CSII-JDP2		
	EdU +	Total	Growing cells(%)	EdU +	Total	Growing cells(%)
#1	182	449	40.5	245	456	53.7
#2	171	487	35.1	245	516	47.5
#3	180	453	39.7	260	501	51.9
#4	201	511	39.3	219	458	47.8
#5	191	475	40.2	277	548	50.5
#6	163	450	36.2	190	399	47.6
Av.			38.5			49.8
SD			2.3			2.6
p	$1.2X10^{-5}$					

The numbers of growing and total cells for figure 4

plko						
	CSII			CSII-JDP2		
	EdU +	Total	Growing cells(%)	EdU +	Total	Growing cells(%)
#1	252	607	41.5	147	414	35.5
#2	223	582	38.3	120	464	25.9
#3	207	559	37.0	130	454	28.6
#4	283	672	42.1	198	688	28.8
#5	212	598	35.5	184	701	26.2
Av.			38.9			29.0
SD			2.9			3.9
p	$1.8X10^{-3}$					

shp53						
	CSII			CSII-JDP2		
	EdU +	Total	Growing cells(%)	EdU +	Total	Growing cells(%)
#1	177	427	41.5	155	527	29.4
#2	233	469	49.7	132	386	34.2
#3	212	441	48.1	159	447	35.6
#4	264	617	42.8	207	678	30.5
#5	230	518	44.4	130	453	28.7
Av.			45.3			31.7
SD			3.5			3.0
p	$1.7X10^{-4}$					

shRb						
	CSII			CSII-JDP2		
	EdU +	Total	Growing cells(%)	EdU +	Total	Growing cells(%)
#1	309	474	65.2	246	611	40.3
#2	302	458	65.9	200	490	40.8
#3	296	451	65.6	228	559	40.8
#4	312	467	66.8	216	559	38.6
#5	322	514	62.6	223	545	40.9
Av.			65.2			40.3
SD			1.6			1.0
p	$1X10^{-9}$					

shp53+shRb						
	CSII			CSII-JDP2		
	EdU +	Total	Growing cells(%)	EdU +	Total	Growing cells(%)
#1	544	627	86.8	468	547	85.6
#2	737	869	84.8	529	628	84.2
#3	659	773	85.3	473	535	88.4
#4	659	760	86.7	415	479	86.6
#5	615	712	86.4	533	620	86.0
Av.			86.0			86.2
SD			0.9			1.5
p	0.83					

Nakade et al. Supplemental Table 1
